# Host hindrance to HIV-1 replication in monocytes and macrophages

**DOI:** 10.1186/1742-4690-7-31

**Published:** 2010-04-07

**Authors:** Anna Bergamaschi, Gianfranco Pancino

**Affiliations:** 1 Institut Pasteur, Unité de Régulation des Infections Rétrovirales, Paris, France

## Abstract

Monocytes and macrophages are targets of HIV-1 infection and play critical roles in multiple aspects of viral pathogenesis. HIV-1 can replicate in blood monocytes, although only a minor proportion of circulating monocytes harbor viral DNA. Resident macrophages in tissues can be infected and function as viral reservoirs. However, their susceptibility to infection, and their capacity to actively replicate the virus, varies greatly depending on the tissue localization and cytokine environment. The susceptibility of monocytes to HIV-1 infection *in vitro *depends on their differentiation status. Monocytes are refractory to infection and become permissive upon differentiation into macrophages. In addition, the capacity of monocyte-derived macrophages to sustain viral replication varies between individuals. Host determinants regulate HIV-1 replication in monocytes and macrophages, limiting several steps of the viral life-cycle, from viral entry to virus release. Some host factors responsible for HIV-1 restriction are shared with T lymphocytes, but several anti-viral mechanisms are specific to either monocytes or macrophages. Whilst a number of these mechanisms have been identified in monocytes or in monocyte-derived macrophages *in vitro*, some of them have also been implicated in the regulation of HIV-1 infection *in vivo*, in particular in the brain and the lung where macrophages are the main cell type infected by HIV-1. This review focuses on cellular factors that have been reported to interfere with HIV-1 infection in monocytes and macrophages, and examines the evidences supporting their role *in vivo*, highlighting unique aspects of HIV-1 restriction in these two cell types.

## Introduction

Bone marrow-derived monocytes (Mos) are released into the blood where they circulate for a few days (the half-life of circulating Mos in normal healthy individuals is 71 h [1]) before subsequent extravasation into the lungs, gastrointestinal tract, kidney, primary and secondary lymphoid organs and the central nervous system (CNS). In tissues, Mos undergo differentiation into tissue-specific macrophages (Mφ) and dendritic cells (DC). HIV-infected mononuclear phagocytes (bone marrow (BM) and blood Mo, tissue Mφ, microglia, and DC) can thus serve as vehicles for dissemination and reservoirs of HIV-1 infection [2]. In the macaque model, the blood Mo count increases during the first few days following SIV infection [3], and high Mo turnover during SIV infection is a predictive marker for AIDS progression [4]. Subsets of activated Mo that express CD16 and/or CD163 are expanded both in HIV-infected individuals and in SIV-infected macaques [5]. During acute infection, activated Mos migrate into different tissues, including the CNS ([3]*and accompanying review by G. Gras and M. Kaul*). Relatively few Mos in the blood bear HIV-1 DNA (<0.1%) [6], reviewed in [7], whereas Mφ vary greatly in their permissivity to HIV-1 infection depending on their tissue localization [8]. Viral replication in tissue Mφ is probably governed not only by the cytokine network, but also by other environmental factors. *In vitro*, Mφ differentiated from blood Mos (Mo-derived macrophages, MDMs) display a great heterogeneity in their capacities to replicate HIV-1, depending on the donor (up to a 3 log difference in viral production between donors) [9-11]. In contrast, HIV-1 replication kinetics were similar in MDM from pairs of identical twins [9]. These observations strongly argue in favor of the influence of the genetic background on viral replication in Mo/Mφ [12], as has also been suggested for CD4+ T cells [13]. Indeed, the CCR5Δ32 genotype has been associated with a restricted infection of MDM and CD4+ T cells by HIV-1 strains that use the CCR5 co-receptor (R5 HIV-1) [11,14,15]. Thus both constitutive and environmental factors appear to regulate HIV-1 replication in Mo/Mφ. Due to the difficulty of assessing HIV-1 infection in resident tissue Mφ, most studies have addressed the regulation of HIV-1 infection in Mo/Mφ in the MDM model. Methodological differences in the purification and differentiation of Mos therefore add further variability to the heterogeneity of these cells with respect to infection by the virus. Several recent reviews have addressed the influence of cytokines and other endogenous and exogenous stimuli on HIV-1 infection of Mo/Mφ [16-18]*(see also the accompanying review by G. Herbein and A. Varin)*. This review will focus on the mechanisms of HIV-1 restriction in Mo and Mφ. *In vitro *data will be discussed for their potential relevance in the light of our knowledge concerning the *in vivo *infection of these cells.

## Molecular shields against HIV-1 replication in monocytes

**Figure 1:**
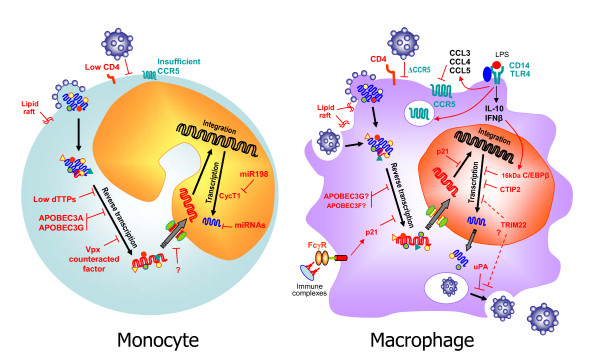
**Schematic representation of host restriction factors in human Mos and Mφ**. On the left, low levels of CD4 and CCR5 may limit viral entry in monocytes. Low expression of thymidine phosphorylase associated with a limited stock of dTTP reduces RT rate. APOBEC3A and 3G may interfere with HIV-1 RT in Mos. HIV-2/SIV Vpx antagonizes the restriction of HIV-1 in Mos and Mφ by counteracting an unidentified host factor. Cellular miRNAs have been proposed to target the 3'UTR of HIV-1 transcripts. miR-198 may repress CycT1 expression that contributes to Tat transactivation. On the right, the CCR5Δ32 mutation restricts viral entry of R5 HIV-1 in Mφ. LPS targets the early phases of the HIV-1 cycle in Mφ, through the down-regulation of CCR5 expression and the LTR-driven transcription by IL-10/IFN-β-induced expression of 16 kDa C/EBPβ. p21^Waf1 ^interferes with both RT and integration and is induced by FcγR engagement. CTIP2 and TRIM22 have been implicated in the inhibition of HIV-1 transcription. Urokinase-type plasminogen activator (uPA) blocks the release of viral particles from intracellular vacuoles.

Although infectious virus can be recovered from peripheral blood Mos taken from HIV-1-infected patients (see below), freshly isolated Mos are highly resistant to HIV-1 infection *in vitro *[19-21]. There are divergent reports on the level of refractivity of freshly isolated quiescent Mos, *in vitro*, to HIV-1 infection, varying from absolute to relative. Methodological parameters including the viral strain and infectious dose, the time of Mo infection after their isolation from blood (immediately or following some hours of culture), the Mo condition at the time of infection (fresh or thawed), and the time lapse of monitoring viral replication after infection, may explain the reported differences in refractivity to HIV-1 replication [22-26]. In addition, the markers used to evaluate Mo differentiation differ depending on the study [24,27,28], and may not completely reflect phenotypic changes associated with maturation. Even when cultured in the absence of human serum or exogenous cytokines such as M-CSF or GM-CSF, Mos may undergo partial differentiation that could modify their capacity to support viral replication [29,30]. Indeed, permissiveness to HIV-1 infection *in vitro *increases with Mo differentiation to Mφ [19,28,31]. The association of Mo maturation with an enhancement of viral replication appears to be a conserved phenomenon among the lentiviruses, as it has also been described for non-primate lentiviruses such as the caprine arthritis-encephalitis virus and maedi-visna virus (MVV) [32,33]. However, while MVV replication in monocytes appears to be restricted at transcriptional level [34,35], distinct mechanisms of restriction contribute to render Mo resistant to HIV-1 infection, at least *in vitro *(Fig. 1A). The relative weight of the restrictions affecting different steps of viral replication is still subject of debate, although pre-integrative blocks appear to play a determinant role.

### Restrictions at early steps of HIV-1 replication in monocytes

The early events of viral entry are represented by the engagement between CD4 receptors at the membrane of target cells and the viral envelope proteins gp120-gp41. The consequent conformational changes in the structure of gp120 allow the interaction with the CXCR4 or CCR5 co-receptors, the latter being the primary co-receptor used by macrophage-tropic HIV-1 strains. Increasing susceptibility of maturing Mos to R5 HIV-1 infection has been associated with an increasing expression of CCR5 at the cell surface that enhances viral entry into the cells [28,36]. However, HIV-1 restriction in Mos does not appear to be due to limiting amounts of HIV-1 co-receptors, and has been attributed to post-entry blocks. Indeed, Mos do not support transduction with HIV-1-based vectors pseudotyped with the VSV-G or MLV-A envelopes, that mediate viral entry by pathways independent of the HIV-1 receptor and co-receptors [27,29], indicating that the block to HIV-1 infection is independent of the route of viral entry. Furthermore, efficient entry of HIV-1 pseudoviruses has been directly demonstrated using a β-lactamase entry assay [27]. Post-entry blocks in infected Mos have been localized either prior to or at the reverse transcription (RT) step of viral replication [26,27] or at the level of nuclear translocation of viral cDNA [29].

A recent study challenges these conclusions, claiming that the relative HIV-1 restriction in Mos, in comparison with Mφ and HeLa-P4 cells, is related to a defect in viral entry followed by a delay in the preintegrative steps [24]. In this work, the inhibition of viral entry into Mos was measured using a fusion assay and was found to be independent of HIV-1 Env, since it also affected VSV-G pseudotyped viruses. Subsequent post-entry steps, RT and integration were not totally blocked, although they proceeded with very slow kinetics (t_IN50% _= 7-8 days) [24]. Neither the nature of the entry block in Mos nor the potential impact of different endocytic/phagocytic capacities of Mos and Mφ with respect to entry of viral particles into cells was addressed in this study.

Using VSV-G pseudotyped HIV-1 and qPCR, Triques and Stevenson showed that reverse transcription is restricted in Mos, and they suggested that the absence of reverse transcription-favouring cellular cofactors is the limiting circumstance [27]. It has been suggested that the defect in reverse transcription observed in Mos, as well as the slow reverse transcription seen in MDMs, is due to a limited availability of nucleotide precursors in these non-dividing cells [37,38]. In particular, Mos contain very low levels of deoxythymidine triphosphate (dTTP), associated with low levels of thymidine phosphorylase, the enzyme that converts thymine into thymidine [27]. Both dTTP and thymidine phosphorylase levels increase during maturation to Mφ. However, D-thymidine supplementation of Mo cultures increased the dTTP levels but did not relieve the reverse transcription block [27], suggesting that other factors are involved in the restriction. In addition, reverse transcriptase from lentiviruses have been shown to be able to efficiently catalyze DNA synthesis even at low dNTP concentrations, in contrast to the RT of gammaretroviruses, which are unable to replicate in non-dividing cells [39].

In contrast to the hypothesis that links Mo resistance to HIV-1 with a lack of cellular cofactors needed for viral replication, Peng et al. proposed that viral replication in Mos is restricted because of factors belonging to the APOBEC3 cytidine deaminase family [40]. The best-characterized member of this family concerning its anti-retroviral activity, including HIV-1 restriction, is APOBEC3G [41-44]. APOBEC3G is incorporated into HIV-1 virions and deaminates dC to dU in minus single-strand nascent cDNA within newly infected cells; resulting in lethal G-to-A hypermutations in the single stranded viral intermediates. This antiviral activity is counteracted by the Vif protein, that induces degradation of APOBEC3G and prevents its incorporation into virions (recently reviewed in [45]). A deaminase-independent anti-viral activity, not counteracted by Vif, has also been described that affects the accumulation of reverse transcripts in infected cells [46,47]. Several mechanisms have been proposed for such antiviral activity, including the inhibition of viral cDNA synthesis by a block in the translocation of reverse transcriptase along the template RNA genome and the destabilization of viral core morphology and stability during virion assembly [47], reviewed in [48]. The APOBEC3G non-enzymatic activity has been proposed to account for the post-entry HIV-1 restrictions in quiescent resting CD4+ T cells [49] and in DC [50], although its role in quiescent CD4+ T-cells has been recently contested [51,52]. The expression of APOBEC3G, and of another member of the same family APOBEC3A, has been shown to be down-regulated during Mo differentiation to Mφ [40]. siRNA-mediated silencing of each of the two genes allowed HIV-1 replication in Mos, whereas induction of APOBEC3A and 3G by IFNs was associated with the inhibition of HIV-1 replication in Mφ [40]. However, the way in which APOBEC3A and 3G interfere with HIV-1 replication in Mos remains to be determined.

Experiments of transduction of heterokaryons formed by the fusion of Mos and permissive HeLa cells with HIV-1 vectors showed that the heterokaryons were refractory to transduction, suggesting the presence of a dominant restriction factor in the parental Mos [53]. HIV-1 restriction in Mo/HeLa heterokaryons could be alleviated by providing the Vpx protein from SIV, either in *trans *or packaged into HIV-1 virions [53]. Vpx has been shown to be required for the replication of HIV-2 and SIV in Mφ, and it has been hypothesized that it diverts a cullin-ubiquitine ligase complex to inactivate a factor that restricts HIV-2 and SIV infection. Vpx expression also enhanced HIV-1 transduction of Mφ, pointing to a common mechanism of restriction [53]. The role of Vpx and the mechanisms underlying its activity in overcoming a retroviral restriction in myeloid cells [54] is discussed in an accompanying review *(Ayinde D. et al.)*.

### Restriction of transcription and later events in HIV-1 replication in monocytes

Besides restrictions at early post-entry steps of viral replication, transcriptional restriction has also been reported to contribute to Mo resistance to HIV-1 [55]. The 5' LTR of integrated provirus contains several cis-regulatory elements necessary for the binding of cellular transcription factors (NFκB sites, C/EBP sites, Sp1 sites and a TATA cassette) and is recognized by the RNA polymerase II as a promoter. The viral Tat protein is recruited to the 5' LTR sequence, interacts with a 59-nucleotide structure called the transactivation response (TAR) element and acts as a stimulator of transcriptional elongation soon after the generation of short terminated transcripts. Tat interacts with the host cyclin T1 protein (CycT1), which recruits the cyclin-dependent kinase 9 (CDK9) to the TAR element. The complex formed by CycT1 and CDK9 is called P-TEFb (for positive transcription elongation factor b). The cooperation of Tat and P-TEFb at the TAR sequence produces a hyperphosphorylation of the C-terminus of RNA polymerase II, stimulating the elongation of viral RNA. After transfection of the HIV-1 genome or of an LTR-reporter construction, neither viral production nor Tat transactivation were detected in undifferentiated Mo [25]. Heterokaryons between Mo and 293 T cells restored the Tat transactivation function of the LTR, suggesting that Mo lack factors required for transactivation. The level of the CycT1 P-TEFb component required for Tat transactivation was below the detection threshold in Mos, in agreement with previous reports [56,57]. The regulation of CycT1 seems to occur at a post-transcriptional level and is likely to involve proteasome-mediated proteolysis [58]. Interestingly, lack of CycT1 expression in Mos has recently been linked to a translational repression by the miR-198 microRNA [59]. It has been proposed that miR-198 contributes to HIV-1 restriction in Mos by repressing CycT1 expression, while miR-198 is down-regulated during Mo differentiation to Mφ [59]. However, transient expression of CycT1 did not rescue Tat transactivation in Mos [25], suggesting that this is not sufficient to relieve HIV-1 transcriptional restriction. Increased permissivity to HIV-1 infection during Mo differentiation to Mφ was associated with both increased expression of CycT1 [25,57] and phosphorylation of the CycT1 P-TEFb partner, CDK9 [25]. It has therefore been suggested that the transcriptional restriction of HIV-1 in Mos may involve regulation of P-TEFb function [25].

Some recent reports have suggested the implication of cellular microRNA (miRNA) in Mo resistance to HIV-1 infection. Wang et al. showed that four miRNA, previously shown to target the 3'UTR of HIV-1 transcripts [60,61], are down-regulated during Mo differentiation to Mφ [62]. This rather preliminary report does not go further into the analysis of miRNA effect on HIV-1 replication. miRNA might target HIV-1 directly or indirectly by side effects on the cell biology [63]. An indirect effect of an miRNA on HIV-1 replication that targets the RNA polymerase II positive transcription elongation factor P-TEFb has indeed been described (see below) [59].

### When HIV-1 meets monocytes *in vivo *...

In spite of the resistance to HIV-1 infection exhibited by Mos *in vitro*, circulating peripheral blood Mos from HIV-1 infected individuals harbor HIV-1 DNA, although at a low frequency (<0.1%) [64,65]. Replication competent virus could be recovered from circulating Mos, even those of patients receiving HAART and with a viral load below detectable levels that would indicate their role as a viral reservoir [66-68]. Compelling evidence for active replication in Mos *in vivo *is supplied by the detection of unintegrated circularized forms of viral DNA (2-LTR circles) and multiply spliced HIV mRNA species in freshly isolated blood Mos [64,68,69], and by markers of compartmentalization and viral evolution in this compartment [70-73].

**Figure 2:**
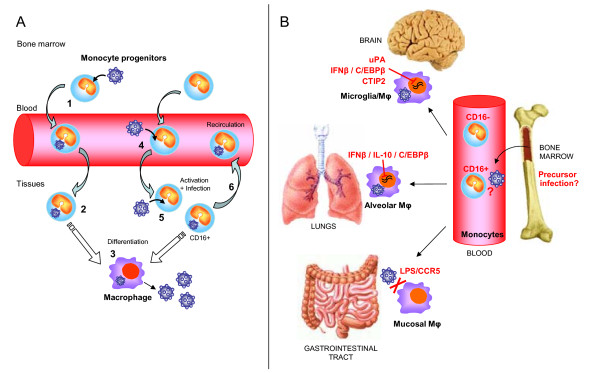
**Schematic model of infection of monocytes and macrophages**. **A) Hypothetical ways to infect monocytes**. Mo precursors may be infected before leaving the bone marrow (1) and then migrate to peripheral tissues where they differentiate into Mφ (2). Viral replication will then be reactivated leading to viral production and infection of neighboring cells (3). Alternatively, Mo subsets may become permissive to infection after being activated in the bone marrow or in the blood, owing to the inflammatory environment (4). Mos may be infected after encountering the virus or infected cells in inflamed tissues (5), where they then differentiate to Mφ. However, infected Mos might also transmigrate back to the blood (6). A Mo subset expressing CD16 that displays pro-inflammatory characteristics appears to be preferentially infected by HIV-1. **B) Dissemination and control of HIV-1 infection in tissue macrophages**. Infected Mos migrate to peripheral tissues such as brain, lungs and gastrointestinal tract where they differentiate and disseminate infection to resident microglial cells, alveolar Mφ or mucosal Mφ. The CD16+ subset has an enhanced capacity to transmigrate into tissues. Various factors that may control HIV-1 replication are present in peripheral compartments. Mφ from the mucosa of the gastrointestinal tract, where exposure to LPS is frequent, do not express CCR5 and are resistant to HIV-1 infection. An increased expression of the inhibitory C/EBPβ may suppress viral transcription in Mφ in brain and lungs, contributing to viral latency. Transcriptional silencing of the HIV-1 LTR by CTIP2 may contribute to HIV-1 latency in the CNS. uPA is also involved in the control of HIV-1 replication in the CNS and is sequestered by the soluble receptor suPAR in CNS disease.

How can observations pertaining to the *in vitro *and *in vivo *contexts be reconciled? It has been suggested that Mos may be infected before leaving the bone marrow (BM) at the stage of precursors, and that they then migrate to other organs, including secondary lymphoid organs, lungs and brain, where they differentiate into Mφ [74] (Fig. 2A). Viral replication will then be reactivated and probably lead to the dissemination of infection to neighboring cells [75] (Fig. 2A). A similar scenario has been hypothesized for MVV infection: infected monocytes carrying the viral genome without expressing viral proteins can enter the organs by a "Trojan Horse" mechanism, avoiding immune surveillance [76,77]. Otherwise, Mo refractivity to HIV-1 may simply not be absolute, and Mo subsets may be permissive to infection. Mos may become permissive to infection after being activated in the BM or in the blood of HIV-1 infected patients, owing to the inflammatory environment and immune activation [78]. Considering the extraordinary plasticity of Mo/Mφ [79], it may also be hypothesized that infected Mos can transmigrate back to the blood [80] after meeting either the virus or infected cells in inflamed tissues (Fig 2A). In support of this possibility, recent evidence has been provided for Mos recirculation from tissues to the BM in a murine model (reviewed in [81]). A subset of circulating Mos that displays pro-inflammatory characteristics is actually expanded in HIV-infected individuals. One minor subset of Mos that expresses the CD16 (FcγRIII) molecule, and represents 5%-15% of circulating Mos in healthy individuals, is expanded in HIV-1 patients and may reach up to 40% of the total circulating Mo population during the progression to AIDS [82]. This Mo subset expresses the CX3CR1 receptor, and its members migrate into tissues that express CXC3CL1, produce pro-inflammatory cytokines (including TNF and IL-1), and can activate resting T-cells by producing CCR3 and CCR4 ligands [83-86]. CD16+ Mos exhibit some characteristics of tissue Mφ and display a transcriptional profile closer to Mφ and DC profiles than to that of CD16- Mos [87-89]. The CD16+ subset of circulating Mos have been shown to be preferentially infected by HIV-1 *in vivo *[90,91] and *in vitro *[90] (Fig 2). Increased susceptibility to R5 HIV-1 was associated with a higher level of CCR5 expression in this cell subset, compared to the CD14highCD16- Mos, and to a shift in the APOBEC3G distribution towards high molecular mass forms [90]. Whether the CD16+ Mos represent a higher level of Mo differentiation and may thus reconcile the findings of Mo restriction to HIV-1 infection *in vitro *and the presence of a fraction of infected Mos *in vivo *remains to be clarified.

## Limits to macrophage permissivity to HIV-1 infection

Mo differentiation to Mφ is accompanied by an increased permissivity to HIV-1 infection, both *in vitro *and *in vivo *(see above). Nevertheless, a great heterogeneity in the capacity to sustain viral replication is observed in MDM from different donors, and HIV-1 infection of resident Mφ varies depending on their tissue localization. In addition, only a fraction of MDM, which varies in size depending on the blood donor, is able to replicate the virus. Some studies suggest that only Mφ which maintain their capacity to proliferate can support a productive HIV-1 infection [31,92]. However, this fact cannot account for differences in the capacity of MDM to replicate HIV-1, since the percentage of cells capable of DNA synthesis is far lower than the percentage of HIV-1-infected cells in MDM cultures [29,93]. Therefore, it appears that HIV-1 replication in Mφ is also regulated by host factors, at the level of both single cells and the individual. Variability in MDM permissivity to HIV-1 infection among individuals has been attributed to host genetic factors that mainly influence pre-reverse transcription steps [12]. The reverse transcription process appears to be the main limiting step of HIV-1 replication, not only in Mo (see above) but also in MDM [10,38,53,94]. However, several other steps of the HIV-1 life cycle that can be restricted in MDM have been described.

### Restrictions at early steps of HIV-1 replication in macrophages

CCR5 co-receptor expression levels at the cell surface are an important determinant for MDM susceptibility to HIV-1 infection. A lack of expression of the CCR5 molecule at the cell surface, linked to a homozygous CCR5Δ32 mutation, blocks the entry of R5 HIV-1 into both CD4+ T cells and MDM [15,95,96]. The heterozygous CCR5Δ32 genotype has also been associated with a decreased susceptibility of MDM to R5 HIV-1 infection [11,14]. A strong and sustained down-regulation of CCR5 expression, independent of *ex novo *protein synthesis but rather due to an altered recycling of chemokine receptors, is induced by exposure of Mφ to lipopolysaccharide (LPS) [97]. This and other mechanisms that underlie LPS-induced restriction of HIV-1 replication in Mφ have been reviewed elsewhere and will not be described here [98]. However, it is worth mentioning that LPS, a major constituent of the cell wall of Gram-negative bacteria, is one of the main stimuli for human Mφ activation and a potent HIV-1 inhibitory factor in these cells that express a wide number of Toll-like receptors (TLRs) and the GPI-anchored CD14 receptor that is responsible for LPS binding. Exposure of Mφ to LPS in physiological conditions might limit viral replication in these cells. In fact, Mφ isolated from the mucosa of the gastrointestinal tract, where exposure to gram-negative bacteria and subsequently to LPS is enhanced, do not express CCR5 at their surface and are resistant to HIV-1 infection [99] (Fig 2B). In addition to the CD4/CCR5 mediated entry of HIV-1 into the cell by membrane fusion, an alternative route of infection has been described in Mφ that involves the uptake of the virus via macropinocytosis [100,101]. This process requires an intact lipid raft, and notably the correct amount and distribution of cholesterol molecules. Cholesterol is a structural component of biological membranes that forms ordered lipid assemblies called lipid rafts, essential for the fluidity of membranes and for the mobility of proteins at the cell surface. Cholesterol may be considered a limiting molecule that can modulate infection by different enveloped viruses, including vaccinia virus, SV40, and herpes simplex virus [102-104]. Chemical cholesterol depletion of target cells has been shown to disrupt HIV-1 entry into primary T lymphocytes and T cell lines as well as into MDM, possibly by reducing the fusion capacity with the HIV-1 envelope and CCR5-mediated CCR5 signaling [105-107].

Mo and Mφ express receptors for the Fc portion of G immunoglobulins (IgG), called FcγR [108]. FcγR molecules form a family of integral membrane proteins that can either activate or inhibit cell functions. The activating receptors expressed on Mφ are the high affinity receptor for monomeric IgG FcγRI (CD64) and two low affinity receptors that only bind the Ag-Ab immune complexes (ICs) FcγRIIA/C (CD32) and FcγRIIIA (CD16). The aggregation of FcγR after binding of IC induces the phosphorylation of their ITAM (immunoglobulin tyrosine activating motif) intracellular activating portion and triggers major responses to pathogens (endocytosis, phagocytosis and cytokine production). FcγRIIB is the inhibitory receptor containing an ITIM (immunoglobulin tyrosine inhibitory motif) in its intracytoplasmic tail, which negatively regulates cell functions induced by the activating FcγR. The stimulation of MDM by IC immobilized on culture plates through activating FcγRs strongly inhibits HIV-1 replication independently of the use of the CXCR4 or CCR5 co-receptors [109,110]. Using one cycle infections, we showed that HIV-1 entry and post-integration steps of the viral replication are not affected in IC-activated MDM, whereas levels of reverse transcription products and integrated proviruses are strongly decreased [110]. Remarkably, other lentiviruses, such as HIV-2, SIVmac and SIVagm, are affected by FcγR engagement, suggesting that the restriction targets either a protein conserved among these viruses or a common function. Recent work showed that the cyclin-dependent kinase inhibitor p21^Cip1/Waf1 ^(p21) knock-down rescues the replication of HIV-1, SIV_mac _and HIV-2 restoring the levels of reverse transcription products and integrated proviruses in IC-activated MDM [111]. Moreover, p21 silencing also increased HIV-1 replication in unstimulated MDM by enhancing reverse transcription and integration. These results suggest that p21 whose expression is enhanced by FcγR engagement acts as an inhibitory factor of lentiviral infection in macrophages. First described as a cell cycle inhibitor, that blocks cell cycling at the G1/S interface and plays a critical role in the control of cell growth, p21 is also involved in the regulation of apoptosis and differentiation [112-114]. Controversial data have been published in the last few years concerning p21 effects on HIV-1 replication in Mφ and in other cell types. Vazquez et al. reported that p21 enhances HIV-1 infection in Mφ 12-14 days after challenge with the R5 BaL viral strain, and proposed that an increased p21 expression after HIV-1 infection was linked to an accumulation of Vpr in infected cells [30]. The reasons for the contrasting findings reported by Vazquez and by ourselves are unclear. They might underlie a dual role for p21 in HIV-1 infection depending on the time after infection: a block of preintegrative steps of HIV-1 replication in acute infection, or an activation of HIV-1 gene expression, synergistically with Vpr, in chronic infection [115]. In T lymphocytes, HIV-1 infection was associated with a loss of p21 expression [116], and 9-aminoacridine (9AA), that induces p21 expression via p53-dependent pathways, significantly inhibits HIV-1 replication in activated PBMCs [117]. p21 was described as a unique molecular barrier for HIV-1 replication in primitive hematopoietic cells that are normally resistant to HIV-1 infection [118]. p21 knockdown in bone marrow CD34+ cells resulted in a strong increase in HIV-1 infection by alleviating a nuclear block to viral genome integration [118]. Zhang et al. showed that p21 was associated with HIV-1 PIC and proposed that the antiviral activity of p21 depends on its ability to interact with HIV-1 integrase (IN). We did not detect interactions between p21 and HIV-1 proteins, including IN, in yeast two-hybrid, pull down or co-immunoprecipitation assays, suggesting that p21 may affect viral replication independently of a specific interaction with an HIV-1 component [111]. Further investigations are needed to precisely determine the interplay between p21 and HIV-1 *(see also data reported in the accompanying review by Le Doucet V. et al)*.

The genetic expression of members of the APOBEC family of cellular polynucleotide cytidine deaminases that have been involved in Mo resistance to HIV-1 infection, including APOBEC3G and APOBEC3A, is down-regulated in Mφ [40]. Besides the species-specific restriction factor TRIM5α [119], an increasing number of TRIM proteins have been found to inhibit several viral infections, including HIV-1 [120]. For instance, TRIM22 (Staf50) has been shown to inhibit HIV-1 replication in MDMs, although its mechanism of action and the step at which the block occurs remain unclear, other than that it appears to affect late steps of HIV-1 replication. Using cell lines, the block has been localized either at the step of viral transcription from the LTR or at that of viral assembly and release [121-123]. TRIM25 participates in RIG-I-mediated antiviral activity through its E3 ubiquitin ligase activity [124]. Although the relevance of antiviral effects of members of the TRIM family has not yet been documented in human Mφ, several TRIM proteins are expressed in these cells and are modulated by external stimuli [125]. Therefore it may be worthwhile to investigate the potential of TRIM proteins for antiretroviral activity. A recent systematic analysis of TRIM gene expression levels in primary human PBMCs and MDM in response to interferons and FcγR engagement may be a helpful tool for further functional studies in this direction [126].

It has been suggested that lentiviruses, unlike other retroviruses, can infect non-dividing cells such as resting T lymphocytes, DC and Mφ, due to the capacity of their cDNA to enter the nuclei through an intact nuclear membrane. A number of mechanisms underlying the interaction of the lentiviral PIC with the cell nuclear import machinery have been proposed to account for this property [127], as reviewed in [128]. In particular, it was proposed that the reduced ability of Vpr-deficient HIV-1 to replicate in MDM reflects the relevance of Vpr-dependent nuclear import in these cells [129]. However, the role of Vpr in the nuclear transport of HIV-1 and in HIV-1 replication in Mφ remains unclear (*see accompanying review, Ayinde D. et al.*). Several studies show redundant nucleophilic determinants in HIV-1 proteins that independently allow the nuclear localization of viral DNA and virus replication in MDM [130-132]. However, a recent study reported that the deletion of all the nuclear localization signals described in HIV-1 proteins did not abrogate HIV-1 infection of resting CD4^+^ T cells and Mφ [133]. The authors proposed that the limiting step that determines the capacity of HIV-1 and MLV to infect non-dividing cells is the uncoating of the entering viral particles, independently of nuclear entry. In the same vein, recent studies concerning HIV-1 and HIV-2 infection of Mφ led to the conclusion that nuclear entry may not be the limiting step for HIV-1 infection, but that the restrictions affect earlier steps before or during reverse transcription [27,134-137].

After passing the nuclear membrane barrier, HIV-1 cDNA is oriented to chromosome targets where viral integrase (IN) catalyzes integration into the host genome [138]. A network of intermediate filament proteins, called lamins, expressed on the inner nuclear membrane ensures the close association between the nuclear envelope and chromatin. The barrier to autointegration factor (BAF), a small DNA-binding protein, is a component of the HIV-1 PIC that promotes integration of the viral cDNA into cell chromosomes and prevents intramolecular integrations. BAF interacts with LEM domain proteins of the inner nuclear membrane (lamina-associated polypeptide 2 (LAP2), emerin, manin). One of its binding partners is emerin, an integral inner-nuclear-envelope protein that participates in chromatin organization and bridges the interface between the inner nuclear envelope and chromosomes. The group of Stevenson proposed that emerin, as well as LAP2α, was required for HIV-1 infection in Mφ to assist the targeting of HIV DNA to the chromatin [139]. The binding of emerin and LAP2α to the viral genome was found to be indirect, and LEM-mediated interaction with BAF was essential to promote integration through the association of these proteins and the viral cDNA. In Mφ that lack emerin or BAF, HIV-1 cDNA entered the nuclear compartment, but was rapidly converted into non-functional episomal DNA that accumulated in the nuclear matrix. Integration into the host genome was therefore dramatically impaired. These results were shortly contradicted by the observation of HIV-1 infection of HeLa-P4 cells following potent down-regulation of emerin, BAF or LAP2α with specific siRNAs [140]. To clarify these conflicting data based on RNA interference-mediated gene knockdown, which were therefore highly dependent on the silencing efficiency, another group demonstrated that HIV-1 efficiently infects embryonic fibroblasts taken from emerin knockout, LAP2α knockout or emerin-LAP2α double knockout mice [141]. The same results were found in Mφ from wild-type and knockout mice transduced with HIV-1, indicating that emerin and LAP2α are dispensable for HIV-1 infection in mouse/human dividing/non-dividing cells. A third experimental approach based on the use of dominant negative emerin molecules presenting mutations in the LEM domain confirmed that HIV-1 infections occur even in the presence of high levels of mutant proteins [141]. Future studies of the role of BAF and its associated nuclear lamin proteins *in vivo *during HIV-1 infection could possibly add further clarification to the interaction of PIC with the nuclear membrane.

### Transcriptional control of HIV-1 in macrophages

Transcriptional regulation has been involved in viral latency of integrated HIV-1 and the formation of viral reservoirs in Mos (see above) and in Mφ. LPS acts as a potent modulator of HIV-1 transcription, displaying opposite effects on Mos and Mφ. Early reports showed that LPS potently stimulates HIV-1 LTR expression in monocytic cell lines by induction of NFκB [142] and through the activation of PU.1 Ets proteins [143]. LPS induces the phosphorylation of PU.1, which allows its interaction with the LTR promoter [143] and with the NFκB transcription factor bound to the downstream binding site. The ability of LPS to induce or suppress transcription from the HIV LTR is linked to the maturation state of monocytic cells. In freshly isolated Mos, LTR-driven gene transcription is enhanced by LPS stimulation, whereas it is suppressed in MDM [144]. A factor contributing to this dichotomy could be the different expression of CycT1, required for Tat transactivation, that is undetectable in Mos, but is induced during the differentiation to Mφ [57] (see above). Although CycT1 is later down-regulated in differentiated MDM, its expression is enhanced by HIV-1 infection [58].

Further insight pertaining to the dual effect of LPS on HIV-1 gene expression came from the observation that LPS modulates the expression of the CCAAT enhancer binding protein β (C/EBPβ) transcription factors differently in Mos and in Mφ [145,146]. C/EBPβ is a member of the C/EBP transcription factor family that is associated with myelomonocytic differentiation [147]. C/EBP binding sites are required for the control of viral replication in Mo/Mφ but not in T lymphocytes [148,149]. Three C/EBP binding sites are localized upstream of the transcriptional start site within the HIV-1 LTR [150]. The C/EBPβ gene has no introns. However, two different proteins can originate from the same mRNA: a large isoform of 30-37 kDa that stimulates gene transcription, and a small isoform of 16-21 kDa that has repressive activity [151,152]. The small inhibitory form of the protein is produced when an internal ribosome entry site is used by ribosomes to start translation [151]. The 16 kDa C/EBPβ protein can be considered to be a dominant negative transcription factor since it blocks DNA transcription even when it is expressed at relatively low levels (20%) compared to the 37 kDa activating isoform [151]. The complex regulation pattern of HIV-1 gene expression by C/EBPβ in Mos and Mφ has been addressed in a series of studies by M. Weiden et al. concerning HIV-1 replication in lung Mφ during pulmonary tuberculosis [145,153-155]. In HIV-1 infected patients, alveolar Mφ (AM) do not show active viral replication, whereas they represent a major source of virus in pulmonary tuberculosis [156]. The inhibitory 16 kDa C/EBPβ isoform is highly expressed in resting AM of healthy individuals, and may be responsible for viral latency in these cells after HIV-1 infection, but it is strongly suppressed after *M. tuberculosis *infection [153]. However, *in vitro *infection with *M. tuberculosis *or stimulation of PMA-differentiated THP-1 monocytic cells and primary Mφ with LPS did not enhance HIV-1 infection, and even suppressed viral replication [145]. In contrast, *M. tuberculosis *and LPS enhanced HIV-1 replication in undifferentiated THP-1 monocytic cells. These opposing effects were reflected by significant changes in the C/EBPβ isoform balance upon exposure to *M. tuberculosis *and LPS in Mos and Mφ: a high amount of activating C/EBPβ transcription factor was induced in Mos, whereas a strong expression of the inhibitory 16 kDa form was induced in Mφ. It turned out that the production of the dominant negative C/EBPβ isoform is mediated by IFNβ in Mφ but not in Mos [145]. LPS and *M. tuberculosis *trigger IFNβ production in both Mo and Mφ. However, while in Mφ IFNβ induces inhibitory C/EBPβ gene expression by stimulating the nuclear translocation and the DNA binding of ISGF-3 (a heterotrimeric complex formed by the interferon regulatory factor IRF-9, STAT-1 and STAT-2), these two stimuli are not sufficient to activate ISGF-3 in Mos [145]. LPS or *M. tuberculosis*-derived lipoarabinomannan induction of IL-10 can also trigger the production of the inhibitory C/EBPβ in differentiated THP-1 Mφ, but not in undifferentiated Mos, through STAT-3 signaling [155]. Thus, differentiation-induced post-translational regulations govern the production of inhibitory C/EBPβ in response to either IFNβ or IL-10 in Mφ. An explanation for the apparent discrepancy between the *M. tuberculosis*-mediated HIV-1 suppression in Mφ *in vitro *and the enhancement of HIV-1 replication in AM *in vivo *was proposed in another study by the same group [154]. The addition of activated T lymphocytes to AM reduced inhibitory C/EBPβ and activated the NF-κB pathway, leading to activation of the HIV-1 LTR and increased viral replication. Down-regulation of inhibitory C/EBPβ expression and subsequent de-repression of the HIV-1 LTR were mediated by the interaction of T cell-expressed co-stimulatory molecules, including CD40L, VLA-4 and CD28, and the cognate macrophage-expressed ligands. The induction of NF-κB was mediated by cytokines secreted from activated T-cell, including TNFβ, IL-1β and IL-6 [154]. Erythromycin A derivatives counteract the positive effect of CD4+ T cells on HIV-1 replication in resistant Mφ by blocking MAPK activation and C/EBPβ induction [157]. Moreover, erythromycin A derivatives render tissue Mφ resistant to HIV-1 infection by inducing the inhibitory C/EBPβ isoform and by down-regulating the activity of hematopoietic cell kinase (Hck) [157]. Recently, IFNβ was shown to induce the truncated inhibitory C/EBPβ isoform and to suppress SIV replication in primary Mφ of rhesus macaques [158]. A downstream effector of class I IFNs, CUGBP1 (CUG-repeat RNA-binding protein 1), was shown to induce the expression of the inhibitory C/EBPβ form by alternative translation of its mRNA [158]. Indeed, the inhibition of SIV replication and the increase of 16 kDa C/EBPβ by IFNβ were associated with and dependent on the phosphorylation of CUGBP1 and the formation of CUGBP1-C/EBPβ mRNA complexes.

A distinct mechanism of HIV-1 transcriptional repression was described in a human microglial cell line. A co-repressor known as the COUP-TF interacting protein 2 (CTIP2) potently inhibited Tat transactivation, and over-expression of CTIP2 disrupted Tat nuclear localization and its recruitment to CTIP2-induced nuclear structures [159]. The authors proposed that Tat inactivation occurs through subnuclear relocalization within inactive regions of the chromosomes [159]. CTIP2 inhibited Sp1- and COUP-TF-mediated activation of HIV-1 gene transcription in microglial nuclei [159]. Indeed, CTIP2 was recruited to the HIV-1 LTR promoter via its interaction with Sp1 bound to the GC-box sequences. CTIP2 co-localized with Sp1, COUP-TF and the heterochromatin-associated protein HP1α that is normally detected in transcriptionally repressed heterochromatic regions [160]. In addition, HDAC1, HDAC2 and the histone methyltransferase SUV39H1 were recruited to the chromatin by CTIP2 and promoted the association of HP1 to the HIV LTR region, thereby silencing viral gene transcription. CTIP2 thus induces HIV-1 gene silencing by forcing the transcriptionally repressed environment onto the LTR promoter [160]*(see also the accompanying review by Le Douce V. et al.)*.

### Restriction of late events in HIV-1 replication in macrophages

HIV-1 assembly in infected Mφ occurs within intracellular compartments associated with the tetraspanin proteins CD63, CD81, CD9 and CD53 [161]. The nature of these vesicular structures is uncertain, some authors claiming that they belong to the system of late endosomes/multivesicular bodies (LE/MVB), others that they represent deep invaginations of the plasma membrane (reviewed in [107], *see also the accompanying review by Benaroch P. et al.*). Virions that have accumulated in these vesicles can be released into the extracellular fluid either directly or after fusion with the plasma membrane, depending on the hypothesis invoked. Urokinase-type plasminogen activator (uPA) signaling has been shown to inhibit a post-translational step of HIV-1 replication in MDM by promoting the sequestration of HIV-1 particles in intracellular vacuoles, possibly related to MVB, which affects the maturation and release of HIV-1 from infected cells [162,163]. uPA is a serine protease that interacts with a specific GPI-anchored receptor, uPAR (CD87), at the cell surface [164]. uPAR is expressed by inflammatory cells including T cells, Mos and Mφ, and regulates cellular functions such as adhesion, proliferation and activation. The interest in the uPA/uPAR system in AIDS has risen from the observation that in a cohort of HIV-1 infected patients, the serum level of the suPAR soluble receptor was closely correlated to the mortality rate before anti-retroviral treatment and was an independent predictor of survival [165]. uPAR expression is up-regulated *in vivo *and *in vitro *by HIV-1 infection [166,167]. In 2001 an HIV-1 suppressor factor was identified from the culture supernatants of an immortalized CD8+ T cell clone [163]. This factor corresponded to the amino-terminal fragment (ATF) of uPA. Urokinase can be found as two enzymatically active isoforms, a high molecular weight form (HMW-uPA) and a low molecular weight form (LMW-uPA) that lacks 135 amino acids of the N-terminus tail of the HMW-uPA. The 135 amino acid peptide, which is naturally cleaved during the processing of HMW-uPA, corresponds to ATF and is catalytically inactive. Late steps of viral replication, such as budding or viral particle assembly, are affected by ATF [163,168]. Similarly, uPA inhibits HIV-1 replication in primary MDM, lymphocytes and monocytic cell lines. The uncleaved inactive precursor of uPA, pro-uPA, which interacts with the same membrane receptor, also inhibits the replication of HIV-1 in MDM, activated PBMCs and *ex vivo *cultures of lymphoid tissue that have been infected *in vitro *[162,168]. The uPA-uPAR interaction interferes with late events of HIV-1 replication in MDM and U937 pro-monocytic cells, as well as in PMA- and TNFα-differentiated U1 cells, inhibiting the release of virions from cells [162,163,168]. The association of the receptor with other signaling competent receptors was required for this inhibitory activity. In particular, the engagement of β1 and β2 integrins, as well as Mac-1 integrin bound to fibrinogen, was identified as mediator of the uPA antiviral effect [168]. Interestingly, cross-linking of Mac-1 also inhibited viral replication. Signaling from uPA/uPAR interaction and assembly of Mac-1 are thus able to interfere with virion assembly and release in Mφ independently of uPAR [168]. The interaction of uPAR and integrins may occur at the level of lipid rafts of the plasma membrane that have been previously described to be a limiting factor for viral entry and budding (see above) suggesting an additional role of these structures in the accumulation/release of viral particles from infected cells.

### Regulation of HIV-1 infection in tissue macrophages

Resident Mφ in tissues are heterogeneous in terms of phenotype, morphology and function [169]. Their characteristics probably depend on the specific tissue microenvironment, as well as on the conditions of inflammation during infections. Accordingly, Mφ from different tissues, such as lung, brain, gastrointestinal and genital tracts, while comprising many HIV reservoirs, display different susceptibilities to HIV infection (Fig. 2B).

Two reports concerning the use of cervical and vaginal explants and purified cell populations from vaginal mucosa provided evidence that subepithelial Mφ are susceptible to infection with monocytotropic R5 HIV-1 strains, and suggested that these cells may represent the main target for HIV infection in the female genital tract [8,170]. In contrast, jejunum intestinal Mφ did not support viral replication [8]. The basis for this differential permissiveness to HIV-1 infection was related to differences in the expression of the CCR5 co-receptor. Mφ from the vaginal mucosa display a similar phenotypic profile to that of blood Mos, and express CD4 and CCR5, whereas Mφ from the jejunum intestinal mucosa express a distinct phenotype, with very low levels of CD4 and virtually no CCR5 [8,171,172]. Therefore the latter cells could resist HIV-1 infection by restricting viral entry due to a lack of the CCR5 co-receptor or to an inappropriate CCR5/CD4 stochiometry [28,173] (Fig. 2B). Susceptibility of Mφ in the intestinal mucosa to HIV-1 infection may however vary depending on their localization at different sites of the intestinal tract, for example in the jejunum *versus *in the rectum, as well as according to the level of local inflammation. Indeed, HIV-1 and SIV infected CD68+ Mφ are found in the colon mucosa of HIV-infected patients or SIV-infected macaques respectively [174,175].

The main cells infected by HIV-1 in the CNS are perivascular Mφ and resident microglial cells, and in the lung are AM [19,156,176-182] (Fig. 2B). However, although HIV-1 entry into the CNS occurs during acute infection [183,184], viral RNA is almost undetectable during the asymptomatic phase of infection. Few AM in bronchoalveolar lavages (BAL) from HIV-1 infected patients harbor viral DNA, and low genetic variability in viral sequences argues against active viral replication [185]. However, in spite of low or undetectable HIV-1 RNA levels in AM in infected patients, viral replication could be reactivated by stimulation of AM from BAL *in vitro *with granulocyte/macrophage colony-stimulating factor (GM-CSF) and TNF-α, or with *M. tuberculosis *and its purified protein derivative [186,187]. More importantly, reactivation of latent HIV-1 replication in AM occurs during co-infections, including those with opportunistic pathogens such as *M. avium *and *Pneumocystis carinii *[156]. Longitudinal studies showed that SIV infection is established in brain and lungs of infected macaques already in acute infection, but HIV-1 replication is then rapidly controlled, and viral RNA becomes undetectable [188,189]. These data suggest that HIV/SIV infection of Mφ in brain and lung is mostly latent before the onset of symptomatic disease, due to the suppression of viral replication. A unifying hypothesis that accounts for the suppression of viral replication in brain and lung has been proposed by Clements et al. Acute HIV/SIV infection induces the production of IFN-β in brain and lung tissues. IFN-β in turn is proposed to induce alternative translation of C/EBPβ mRNA, switching the balance between activating and inhibitory C/EBPβ isoforms in favor of the inhibitory form that suppresses LTR-driven transcription of viral genes [[189,190]. Several other studies support a role for C/EBPβ regulation in HIV-1 expression in the CNS and in neuropathogenesis [149]. In the lung, pulmonary tuberculosis markedly up-regulates HIV-1 replication in AM by repressing the expression of inhibitory C/EBPβ [153]. Lung infiltration of T lymphocytes leads to the loss of inhibitory C/EBPβ mediated by the interaction of cell surface co-stimulatory molecules and ligands during T cell/macrophage contact [154]. Meanwhile, production of pro-inflammatory cytokines by activated T lymphocytes boosts infection by triggering NF-κB activation [154]. It is conceivable that a similar scenario may also occur in brain and other tissues of HIV-infected individuals. Indeed, although HIV-1 infects the CNS during acute primary infection, active viral replication is observed after the loss of immune control, and this coincides with increased immune activation (reviewed in [191]). Reactivation of viral replication from latently infected microglia may occur concomitantly with trafficking of activated Mo/Mφ into the CNS [192]. Interestingly, enteropathy in chronically SIV infected macaques has also been associated with increased expression of the activating isoform of C/EBPβ localized predominantly in Mφ in the jejunum and colon mucosa [175]. Thus, increased expression of activating C/EBPβ may contribute to the maintenance of inflammation and the activation of viral replication in Mφ of different organs. However, other mechanisms of transcriptional silencing of the HIV-1 LTR that have been described in microglial cells may contribute to HIV-1 latency in the CNS [160].

The deregulation of uPA and its receptor uPAR has also been implicated in the loss of control of HIV-1 replication in the CNS [193]. uPA signaling through uPAR inhibits late steps of HIV-1 replication [162,168] (see above). High levels of expression of uPAR were detected in HIV-infected microglial cells and reactive Mφ in the brain of patients with encephalitis and other HIV-related neurological lesions, whereas uPA was detected in few cells [193-195]. In addition, higher levels of the soluble form of uPAR (suPAR), uPA and suPAR/uPA complexes were found in the cerebrospinal fluid (CSF) in HIV-infected patients than in HIV-negative controls, and in patients with ADC or opportunistic CNS infections than in neurologically asymptomatic patients. suPAR levels correlated with CSF HIV-1 RNA [193]. It has thus been proposed that HIV-1 infection induces over-expression of uPAR and consequently overproduction of suPAR. The excess suPAR in the CSF would bind most of the extracellular uPA, preventing its binding to cell surface uPAR and its signaling-induced inhibition of HIV-replication [193].

## Conclusions

The list of putative mechanisms of control of HIV-1 infection in Mo/Mφ is rapidly growing (Table 1). HIV-1 replication is restricted at different steps. More research is, of course, needed to gain further insights into the molecular mechanisms underlying each restriction event. However, importantly, evidence for the relevance of some of these mechanisms *in vivo *is now coming from studies concerning HIV-1 infected individuals and from the SIV/macaque model, as reviewed above for the IFNβ/C/EBPβ or uPA/uPAR pathways. The contribution of other mechanisms of HIV-1 restriction that have been identified *in vitro *to the control of Mo/Mφ infection *in vivo *is still uncertain and requires further studies. For example, whether and how the balance of the inhibitory and enhancing effects of p21 influences the replication of HIV-1 in tissue Mφ remains an open question. The answer to this question may be directly relevant to therapy: modulation of p21 expression is currently studied in anticancer therapy. The role of the restriction factors of the TRIM or the APOBEC3 family in HIV-1 infection of Mo/Mφ is unclear. In particular, APOBEC3A and 3G are strongly induced by IFNs in Mφ [40,196], which is in favor of their antiviral role. Whether and by which mechanisms these molecules contribute to the control of HIV-1 infection in the cells remains to be elucidated [52,196].

**Table 1:** Restrictions that limit HIV-1 replication in monocytes and macrophages.

Monocytes		
**Restriction mechanism**	**Replication step affected**	**Ref.**

APOBEC3G APOBEC3A	Reverse transcription	[40]
Vpx counteracted factor	Post-entry Reverse transcription	[53]
miRNA-198	Transcription (down-modulation of CycT1)	[59]
miRNAs	Viral mRNA	[62]

**Macrophages**		

**Restriction mechanism**	**Replication step affected**	**Ref.**

CCR5 down-regulation, Δ CCR5	Entry	[15,95-97]
p21	Reverse transcription Integration	[111,118]
16 kDa C/EBPβ	Transcription	[145,153-155]
CTIP2	Transcription	[159]
TRIM22	Transcription? Budding and release?	[121-123]
PPAR	Post-integration (?)	[204,205]
uPA	Post-translation, release?	[162,163,168]

New high throughput screening techniques are being used to discover host molecules relevant to HIV-1 replication. Microarray analyses have revealed alterations of gene expression in Mo/Mφ that are associated to HIV-1 infection (reviewed in [197]), but they have not allowed the identification of new molecules involved in the control of HIV-1 replication. Genome-wide screenings, including small interfering RNA (siRNA) screening have provided a huge amount of information [198-201]. However, while they have a high potential to identify host cofactors required for HIV-1 replication and to drive the search for host targets for HIV therapeutics, such approaches may be less suited to unveil factors that inhibit viral replication [201]. For example APOBEC3G was not detected by RNA interference screens [198]. In addition, some factors may act differentially depending on the cell type (for example, the restriction overcome by Vpx in Mos/Mφ and DC). Only studies focused on the relevant cell type will be able to detect these factors.

Last but not least, a question about the interplay between host restriction mechanisms and viral infection is: what is really the ultimate effect of the host factors that hinder HIV-1 replication in host cells and are thought to exert an antiviral activity beneficial to the host. It may be that an effective mechanism of restriction that blocks viral replication in a cell may be subverted by the virus at the organism level, in the complex interplay between the virus and the host. For example, while inhibitory C/EBPβ is an effective anti-viral lock that suppresses HIV-1 transcription, it also allows the virus to remain latent in the brain and lung until the host immune response is declining, and the infection is then unlocked when immune activation boosts viral replication. This could be a conserved strategy of persistence in lentiviral infections: a post-transcriptional block also restricts MVV replication in macrophages in the CNS and lung macrophages unless inflammatory lesions promote viral expression [202,203]. Therapeutic strategies based on restriction mechanisms may therefore be aimed either at enhancing some restriction mechanisms to limit infection or to thwart other events that would otherwise reactivate viral replication and drain viral reservoirs.

## Competing interests

The authors declare that they have no competing interests.

## Authors' contributions

AB and GP were responsible for drafting and revising the manuscript, as well as organizing the content and editing the manuscript. GP conceptualized the figures. AB created Figures 1 and 2 and their legends. Both authors read and approved the final manuscript.
